# Renal Dropsy Following Scarlatina—Suppression of Urine and Convulsions

**Published:** 1869-04

**Authors:** N. S. Davis

**Affiliations:** Professor of Principles and Practice of Medicine in Chicago Medical College, and of Clinical Medicine in Mercy Hospital


### CHICAGO MEDICAL EXAMINER.

N. S. DAVIS, M.D., Editor.

VOL. X. APRIL, 1869. NO. 4.

### Original Contributions.

ARTICLE XV.

### RENAL DROPSY FOLLOWING SCARLATINA—SUPPRESSION OF URINE AND CONVULSIONS.—CLINICAL CASES.

By N. S. DAVIS, M.D., Professor of Principles and Practice of Medicine in Chicago Medical College, and of Clinical Medicine in Mercy Hospital.

There are but few active practitioners who have not found the sequelæ of scarlet fever among the most obstinate and serious ailments that come under their observation.

Among these, none are more important than the renal affections accompanied by anasarca.

As illustrative of the important series of pathological phenomena developed in the progress of such cases, the two following cases are placed on record:—

Case I. C. K., a girl aged twelve years, previously in good health, was attacked with scarlatina simplex during the last week in December, 1868. Two or three other children in the family had the disease at the time, in one of whom it presented the anginose variety, and I was called to prescribe for it. At the time of my visit to the little boy, the girl was sitting up in the same room, complaining so little that the mother did not think she required medical treatment.

The rash had been well developed on the surface, the fever moderate, and the case free from any apparent complications. She convalesced, and appeared well until the 15th of January, when her face and limbs began to swell. On the 16th I was called to see her, and found the surface generally pale, and swollen from anasarca; pulse moderately accelerated; skin dry, and little above the natural temperature; head light, or giddy; dull pain in the back, with sense of heaviness and lameness in bending the trunk on the pelvis; bowels inactive; stomach nauseated at times; appetite much impaired; and urine scanty and dark-colored.

On examination, the latter was found to contain a few blood-corpuscles and much albumen, with epithelium, and fibrinous shreds. She was directed to have a saline laxative to open the bowels, to be followed by a prescription containing digitalis, nitrous ether, and iodide potassa, to be taken every three hours.

On the 18th, I saw her again. Her bowels had moved freely, and she had taken the medicine regularly, but there was no marked change in her symptoms.

The urine had not increased in quantity, and was more bloody. From this to the evening of the 22d, she took freely of bitartrate of potassa dissolved in water; continued the digitalis and nitrous ether, and a powder of nitrate potassa, calomel, and Dover's powder at night.

Warm fomentations were also applied both to the loins and abdomen. The anasarca, however, continued slowly to increase; the pulse became smaller and more frequent; the head more dizzy; the stomach more irritable, and the urine more scanty, and more largely mixed with blood. During the 21st and 22d, she passed not more than three or four ounces in the twenty-fours, and it was about half blood.

On the night of the 22d, she was seized with severe general convulsions. Living in a part of the city distant from my residence, a physician in the neighborhood was called in, who ordered a warm bath, followed by fomentations, and the liberal internal use of bromide and iodide of potassa. The convulsions, however, continued to recur at short intervals, until I saw her on the afternoon of the 23d.

The urinary secretion had been entirely suppressed for the preceding twenty-four hours; the face and whole exterior surface of the body and limbs were much bloated; the pulse 130 per minute, small and weak; skin moist, but temperature nearly natural; pupils dilated; and mind incapable of being roused to consciousness. Warm applications to the trunk and lower extremities were continued, and the following prescription ordered:—

℞. Hydrarg. Chlorid. Mite, ---------------- 20 grs.
Nitras Potassa, -------------------------- 30 grs.

Mix, and divide into 4 powders, one to be taken every two hours, until the bowels are freely moved; the operation of the same to be aided by warm salt-water enemas. To act as temporary antispasmodics, ten drops each, of chloroform and fluid extract of Cannibis Indica, were given between the powders. After she had taken three of the powders and one or two enemas, the bowels began to move freely, and the convulsions ceased. Soon after, she passed four or five ounces of turbid urine.

During the night of the 23d, and morning of the 24th, she urinated two or three times, and the bowels were evacuated copiously, but without attention on her part. She was quiet during the night, and at my visit at 10 A. M. of the 24th, she could be aroused to partial consciousness, but was very feeble. She was ordered a solution of bitartrate of potassa and gum-Arabic for a drink, and a powder containing five grains of nitrate potassa and three grains of Dover's powder, every three hours. All other remedies were dispensed with, except beef-tea for nourishment.

From this time the urinary secretion continued to improve in quantity and quality; the skin continued moist; the pulse became slower and more full; and the mental faculties regained their activity. On the 29th, she had progressed so far in convalescence that she needed no further attendance.

CASE II. Miss M., a girl aged 14 years, usually in good health, was attacked with scarlatina simplex January 11th, 1869. The disease ran a very mild course, requiring but little medical treatment. By the 22d, she seemed quite well again. On the 8th of February was again called, and found her presenting the appearance of a moderate degree of general anasarca, with a dull pain in the loins and head, and scantiness of urine, but no fever. She was directed to have two drachms of the bitartrate of potassa and three drachms of the acetate, dissolved in half a pint of water, and to take a tablespoonful of the solution every four hours. I did not see her again until the 13th, when I was called in great haste. During the preceding night she had been attacked with violent general convulsions.

The prescription made on the 8th had appeared to have very little effect. The bloating of the whole surface had steadily increased, with increase of the pain in the head and back, frequent nausea, and some fever. The urinary secretion had decreased until the 12th, when it became entirely suppressed, and convulsions began the night following. The convulsive paroxysms followed each other in quick succession, allowing only an imperfect degree of consciousness to be recovered between them; the stomach promptly rejected all drinks by vomiting; the pulse was small and frequent; skin cool; pupils slightly dilated; respiration short, but regular, between the convulsive paroxysms. No passage of urine or fæces during the preceding twenty-four hours. A physician had been called in soon after the convulsions commenced, late in the evening of the 12th, who gave the bromide and iodide of potassa freely, but without any perceptible effect. At my visit on the morning of the 13th, I ordered extensive warm fomentations, with a view of promoting the action of the skin, and gave internally a powder of calomel, 5 grains, and nitrate of potassa, 5 grains, every two hours, with 10 drops each of chloroform and fluid extract of Cannabis Indica between. The latter was given more to prevent vomiting than for any supposed antispasmodic influence. The convulsions continued to recur through the day, but at longer intervals; otherwise, there was no improvement in her symptoms, and no evacuations either of urine or fæces. The further use of the powders was suspended, and in their place Croton oil, suspended in the form of an emulsion, was given, in doses of little less than one drop every hour, until evacuations should occur. When she had taken three drops, a part of which was rejected by vomiting, it began to operate freely upon the bowels. On the morning of the 14th, found her quiet; no convulsions since the evening previous; face, and surface generally, still much bloated from dropsical infiltration; skin cool; pulse small, and 120 per minute; mind dull, but capable of being partially roused to activity; and indications of partial paralysis of the left side.

The bowels had been evacuated freely several times, and she had passed a moderate quantity of urine twice. She was directed to have beef-tea in small quantities for nourishment, and a solution of bitartrate potassa for drink. On the 15th, she was much improved in all respects, except the left arm was completely paralyzed, and the movements of the bowels had continued frequent, with some tenesmus and mucus in the discharges.

The urine was nearly natural in quantity and appearance. She complained of some pain in the paralyzed arm, between the shoulder and elbow.

The following prescriptions were ordered:—

℞. Fl. Ext. Scutellaria, ---------------------- ℥iij.

Tinct. Digitalis, --------------------------- ʒj.

Iodide Potassa, ---------------------------- ʒiij.

Mix. Dose, one teaspoonful in sweetened water every four hours. Also,

℞. Ol. Terebinth., -------------------------- ʒij.

Tinct. Opii, -------------------------------- ʒij.

Pulv. G. Acacia, } āā, ------------------- ʒiij.

Saccha. Abla., }

Rub together, and add

Spts. Nit. Dulce, ------------------------ ℥iss.

Mint-Water, ------------------------------- ℥iss.

Mix. Dose, one teaspoonful every four hours, alternating with the other prescription, until the dysenteric irritation of the lower bowel ceases.

The use of the latter prescription was required only three or four times, and the patient improved steadily from day to day, until the anasarca, the paralysis, and nearly all symptoms of disease had disappeared. From the 18th to the 24th, the patient was cheerful and active in mind, appetite good, bowels regular, strength improving, and all the appearances of returning health. But there remained some anasarcous puffiness of the face and lower extremities, and the urinary secretion was unsteady. Some days it was natural in quantity and appearance, others smaller and turbid.

She was confined to the use of light food, subjected to no injudicious exercise or exposure to atmospheric changes, and during the period last named took only mild diuretics and tonics.

Yet, on the 25th, she became dull, more anasarcous, and the stomach irritable, with only a slight discharge of very high-colored urine; and before the next morning convulsions again came on as violent as in the first attack. The same means were resorted to as in the first attack, with the addition of a vapor-bath, and the omission of Croton oil; the powders of calomel and nitrate of potassa operating freely. The convulsions again ceased on the procurement of free intestinal evacuations and the return of renal secretion.

But her subsequent progress to recovery has been very slow and vacillating, and is yet imperfect.

In a practice extending over a period of more than thirty years, during which I have seen a fair proportion of scarlet-fever patients, and have often seen some degree of renal dropsy as a sequel, the two foregoing are the first cases that have occurred in my own practice, of complete suppression of urine and convulsions following this much-dreaded fever. Both these cases occurred after the mildest grade of fever, and in spite of some directly preventive treatment. And neither seemed to be benefited by any remedies except such as aided in the elimination of the retained elements of urine, by promoting the action of the skin, kidneys, and bowels.

